# Predictivity of daily gait speed using tri-axial accelerometers for two-year incident disability among Japanese older adults

**DOI:** 10.1038/s41598-022-14304-9

**Published:** 2022-06-16

**Authors:** Naoto Takayanagi, Motoki Sudo, Yukari Yamashiro, Ippei Chiba, Sangyoon Lee, Yoshifumi Niki, Hiroyuki Shimada

**Affiliations:** 1 Tokyo Research Laboratories, Kao Corporation, 2-1-3 Bunka, Sumida-ku, Tokyo, 131-8501 Japan; 2 Department of Preventive Gerontology, Center for Gerontology and Social Science, National Center for Geriatrics and Gerontology, 7-430 Morioka, Obu, Aichi 474-8511 Japan

**Keywords:** Geriatrics, Quality of life

## Abstract

Gait speed is an important indicator of functional decline in older adults. Recently, daily gait speed has been assessed using accelerometers. However, it is unclear whether this parameter can predict the decline in functional abilities. This study investigates whether daily gait speed can be a predictor of incident disability risk as well as in-laboratory gait speed. A sample of 1860 older adults (Male: 728, Female: 1132; 70.1 ± 6.2 years) were instructed to wear accelerometers on the waist. The association between daily gait speed for two weeks and incident disability during a two-year period was analyzed by using the cut-off value for screening prefrailty in the previous study (106.3 cm/s). Furthermore, the associations with in-laboratory gait speed (cut-off value: 100 cm/s), number of steps (cut-off value: 6342.2 steps/day), and incident disability were also analyzed. Cox proportional hazards analysis showed a significant hazard ratio of low daily gait speed (HR, 2.97; *p* = 0.02) comparable to that of low in-laboratory gait speed (HR: 2.53; *p* = 0.01). Conversely, the number of steps had no significant association with incident disability (HR: 1.99; *p* = 0.12). These results suggest that daily gait speed can be a predictor of incident disability risk in older adults.

## Introduction

Currently, the aging rate is increasing worldwide. The population of adults aged 65 years and older is expected to reach 18.3% by 2060^1^. Due to the physical dysfunction or chronic diseases associated with aging^2^, the risk of incident disability is also increasing^3^, which results in a decrease in the quality of life of older adults^4^.The cost of caring for adults with incident disabilities is a burden on society. Although Japan, with the highest aging rate of the global population^5^, has introduced a long-term care insurance (LTCI) system since 2000^6^, maintaining this system has become challenging because of its high demand^7^. However, the maintenance of functional independence during old age is an important factor for both the well-being of individuals and aging society. Therefore, it is essential to assess the decline of functional abilities in older adults to prevent their functional impairment at an earlier stage.

Gait speed is an important predictor of decline in physical function, especially in older adults^8,9^. A recent large cohort study has revealed that older adults with a gait speed slower than 100 cm/s develop disabilities more frequently than those with faster speeds^10^. Traditionally, gait speed has been assessed mainly in laboratory settings (referred to as in-laboratory gait speed) using a stopwatch, a tape measure, or a sheet-type pressure sensor^10,11^. Recently, multiple researchers have reported the estimation algorithms for gait speed in free-living conditions (referred to as daily gait speed) using accelerometers^12–14^. Zijlstra et al.^12^ compared actual gait speed measured by an observer and gait speed estimated by lower trunk acceleration on a 25 m trajectory at three different speed ranges (slow, preferred, and fast) in healthy participants. They reported that the individuals’ estimated gait speeds were within 15% of their actual average speeds, with some exceptions. Similarly, Schimpl et al.^13^ compared actual gait speed measured by a high-end bicycle computer mounted on a perambulator and estimated gait speed by a tri-axial accelerometer placed inside a belt buckle in free-living conditions in healthy participants; they found that an estimation algorithm employing support vector regression performed with a concordance correlation coefficient of 0.93. Furthermore, Soltani et al.^14^ compared actual gait speed and estimated gait speed by a lower-back-worn sensor on a 12 m path across four different conditions (slow, normal, fast, and using walking aids) for older adults and reported a high estimation accuracy (root mean squared error of 10, 18, 15, and 32 cm/s, respectively). Thus, accuracy verification is conducted based on various conditions, such as measurement place, speed range, age of participants, and measurement location on the body.

In addition, several recent studies have reported on the assessment of daily gait speed. Schimpl et al.^15^ reported that the daily gait speed of healthy participants aged 17–65 years decreases with age. Furthermore, Soltani et al.^16^ stated that the estimated usual gait speed (100–160 cm/s) appears consistent with normative values and expected trends regarding age, gender, BMI, and physical activity levels, and the daily gait speed can be used as a reliable indicator of age-related functional decline. These previous studies suggest that daily gait speed may be associated with a decline in physical function as well as in-laboratory gait speed. However, owing to differences in experimental conditions, it is difficult to conclude whether daily gait speed can be a predictor of decline in physical function and in-laboratory gait speed. Interestingly, a study has reported that there was only a weak correlation between these parameters in the same participant^17^. Therefore, it is important to examine the prospective association between daily gait speed and decline in physical function.

A recent study reported a cut-off value for daily gait speed (106.3 cm/s) and number of steps (6342.2 steps/day) measured by waist-worn accelerometers in older adults with prefrailty^18^. Prefrailty is defined as a preliminary stage of frailty associated with various functional impairments^19,20^, and it is potentially an optimal target for clinical and/or behavioral interventions for functional impairment. We hypothesize that by using the cut-off value of daily gait speed for screening prefrailty^18^, it would be possible to screen older adults who are at high risk of future functional decline. If monitoring daily gait speed with an accelerometer can determine the risk of future physical decline of older adults, it would be possible to prevent further deterioration by providing exercise and dietary interventions to older adults at high risk.

The objective of this study is to investigate whether daily gait speed can be a predictor of decline in physical function in older adults as well as in-laboratory gait speed. By using the same waist-worn accelerometer and cut-off value for daily gait speed (106.3 cm/s), which have produced prior findings regarding prediction in older adults for prefrailty^18^, the association between daily gait speed and incident disability during a two-year period was analyzed. Furthermore, to compare daily gait speed to the associations with in-laboratory gait speed (100 cm/s: cut-off value for incident disability^10^), number of steps (6342.2 steps/day: cut-off value for prefrailty^18^), and incident disability were further analyzed.

## Results

Table 1 shows the gait parameters and baseline characteristics of participants with an incident disability during the two years after baseline assessment. During the two-year follow-up period, 42 participants (2.3%) had an incident disability and were certified as needing care or support according to the LTCI criteria.

**Table 1:** Gait parameters and baseline characteristics of participants by incidence of disability during the two years after baseline assessment.

Characteristics	Overall (n = 1860)	Independent (n = 1818)	Incident disability (n = 42)	P value	Effect size
Age, years	70.1 ± 6.2	69.9 ± 6.1	76.8 ± 6.4	< 0.01*	*d* = 1.13
Sex, female, number (%)	1132 (60.9)	1109 (61.0)	23 (54.8)	0.51	*φ* = 0.02
BMI, kg/m^2^	23.4 ± 3.2	23.4 ± 3.2	24.4 ± 4.0	0.05*	*d* = 0.31
Grip strength, kg	28.0 ± 7.6	28.1 ± 7.6	26.3 ± 7.8	0.13	*d* = 0.24
Education, years	11.5 ± 2.4	11.5 ± 2.4	10.7 ± 2.1	0.03*	*d* = 0.33
GDS, score	2.7 ± 2.5	2.7 ± 2.5	3.5 ± 2.8	0.04*	*d* = 0.32
Prescribed medications, number	2.6 ± 2.5	2.6 ± 2.5	4.2 ± 2.7	< 0.01*	*d* = 0.64
Current worker, number (%)	748 (40.2)	737 (40.5)	11 (26.2)	0.09	*φ* = 0.04
Current smoker, number (%)	144 (7.7)	140 (7.7)	4 (9.5)	0.88	*φ* < 0.01
Current alcohol drinker, number (%)	624 (33.5)	613 (33.7)	11 (26.2)	0.39	*φ* = 0.02
Stroke, number (%)	73 (3.9)	69 (3.8)	4 (9.5)	0.14	*φ* = 0.03
Cancer, number (%)	213 (11.5)	205 (11.3)	8 (19.0)	0.19	*φ* = 0.03
Spine disease, number (%)	348 (18.7)	339 (18.6)	9 (21.4)	0.80	*φ* = 0.01
Fractures after age 60, number (%)	193 (10.4)	184 (10.1)	9 (21.4)	0.03	*φ* = 0.05
Osteoarthritis, number (%)	328 (17.6)	318 (17.5)	10 (23.8)	0.39	*φ* = 0.02
Heart disease, number (%)	264 (14.2)	259 (14.2)	5 (11.9)	0.84	*φ* < 0.01
Hypertension, number (%)	842 (45.3)	813 (44.7)	29 (69.0)	< 0.01	*φ* = 0.07
Diabetes, number (%)	223 (12.0)	215 (11.8)	8 (19.0)	0.24	*φ* = 0.03
Hyperlipidemia, number (%)	564 (30.3)	551 (30.3)	13 (31.0)	0.94	*φ* < 0.01
Osteoporosis, number (%)	124 (6.7)	118 (6.5)	6 (14.3)	0.09	*φ* = 0.04
Respiratory disease, number (%)	184 (9.9)	174 (9.6)	10 (23.8)	< 0.01	*φ* = 0.06
Daily gait speed, cm/s	110.3 ± 22.6	110.6 ± 22.5	95.0 ± 18.8	< 0.01*	*d* = 0.70
In-laboratory gait speed, cm/s	117.7 ± 19.5	118.2 ± 19.1	97.0 ± 23.9	< 0.01*	*d* = 1.10
Number of steps, steps/day	6562.0 ± 2993.4	6610.9 ± 2978.7	4446.8 ± 2897.1	< 0.01*	*d* = 0.73

Data are shown as means ± SDs. Unpaired *t* tests or chi-square tests were conducted between groups.

BMI = body mass index; GDS = geriatric depression scale. *p < 0.05, *d* > 0.20, *φ* > 0.10.

The Pearson’s correlation analysis showed that daily gait speed and in-laboratory gait speed (*r* = 0.325, *p* < 0.001), daily gait speed and number of steps (*r* = 0.354, *p* < 0.001), and number of steps and in-laboratory gait speed (*r* = 0.202, *p* < 0.001) were positively correlated, respectively.

Significant differences were found in age (*p* < 0.01, *d* = 1.13), body mass index (BMI) (*p* = 0.05, *d* = 0.31), education years (*p* = 0.03, *d* = 0.33), Geriatric Depression Scale (GDS) score (*p* = 0.04, *d* = 0.32), and number of prescribed medications (*p* < 0.01, *d* = 0.64). For gait parameters, significant differences were found in daily gait speed (*p* < 0.01, *d* = 0.70), in-laboratory gait speed (*p* < 0.01, *d* = 1.10), and number of steps (*p* < 0.01, *d* = 0.73). The incident disability rates of low daily gait speed, low in-laboratory gait speed, and low number of steps were 4.0%, 7.5%, and 3.7%, respectively.

**Figure 1:**
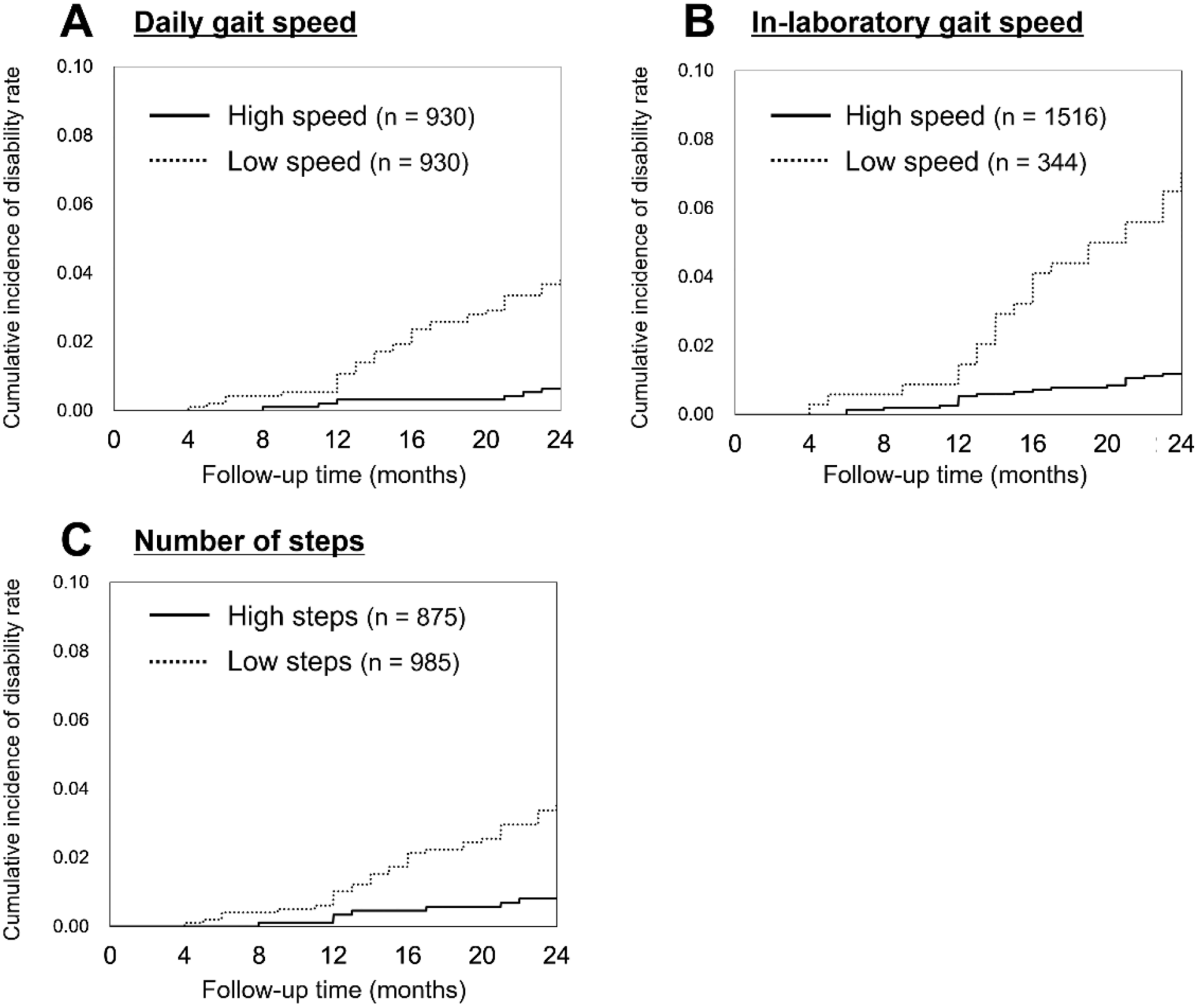
Kaplan–Meier estimates of cumulative incidence of disability according to the three gait parameters (**A**: daily gait speed, **B**: in-laboratory gait speed, **C**: number of steps).

Figure 1 shows the cumulative incidence of disability rate according to the three gait parameters. The Kaplan–Meier log-rank test showed that the incidence of disability rate was significantly higher in the low daily gait speed group than in the high daily gait speed group (*p* < 0.01). Additionally, the incidence of disability was significantly higher in the low in-laboratory gait speed group than in the high in-laboratory gait speed group (*p* < 0.01). Furthermore, the incidence of disability was significantly higher in the low number of steps group than in the high number of steps group (*p* < 0.01).

Table 2 shows the Cox proportional hazards regression models that were used to analyze the associations between the three gait parameters and incident disability risk. In the crude model, the low daily gait speed group showed a significantly higher risk of incident disability than the high daily gait speed group (hazard ratio (HR), 3.71; 95% CI = 1.53–9.02; *p* < 0.01). Similarly, the low in-laboratory gait speed group showed a significantly higher risk of incident disability than the high in-laboratory gait speed group (HR: 4.12; 95% CI = 2.20–7.69; *p* < 0.01). The low number of steps group showed a significantly higher risk of incident disability than the high number of steps group (HR, 2.65; 95% CI = 1.16–6.10; *p* = 0.02).

**Table 2:** Association of gait parameters with disability in the Cox proportional hazard regression model.

	Crude		Adjusted	
	HR (95% CI)	P value	HR (95% CI)	P value
Low daily gait speed (ref: high speed)	3.71 (1.53–9.02)	< 0.01*	2.97 (1.17–7.56)	0.02*
Low in-laboratory gait speed (ref: high speed)	4.12 (2.20–7.69)	< 0.01*	2.53 (1.27–5.01)	0.01*
Low number of steps (ref: high steps)	2.65 (1.16–6.10)	0.02*	1.99 (0.83–4.78)	0.12

HR = hazard ratio; ref = reference, *p < 0.05.

Low and High daily gait speed: participants with daily gait speed of < 106.3 cm/s and of ≥ 106.3 cm/s, respectively.

Low and High in-laboratory gait speed: participants with in-laboratory gait speed of < 100.0 cm/s and of ≥ 100.0 cm/s, respectively.

Low and High steps: participants with number of steps of < 6342.2 steps and of ≥ 6342.2 steps, respectively.

**Figure 2:**
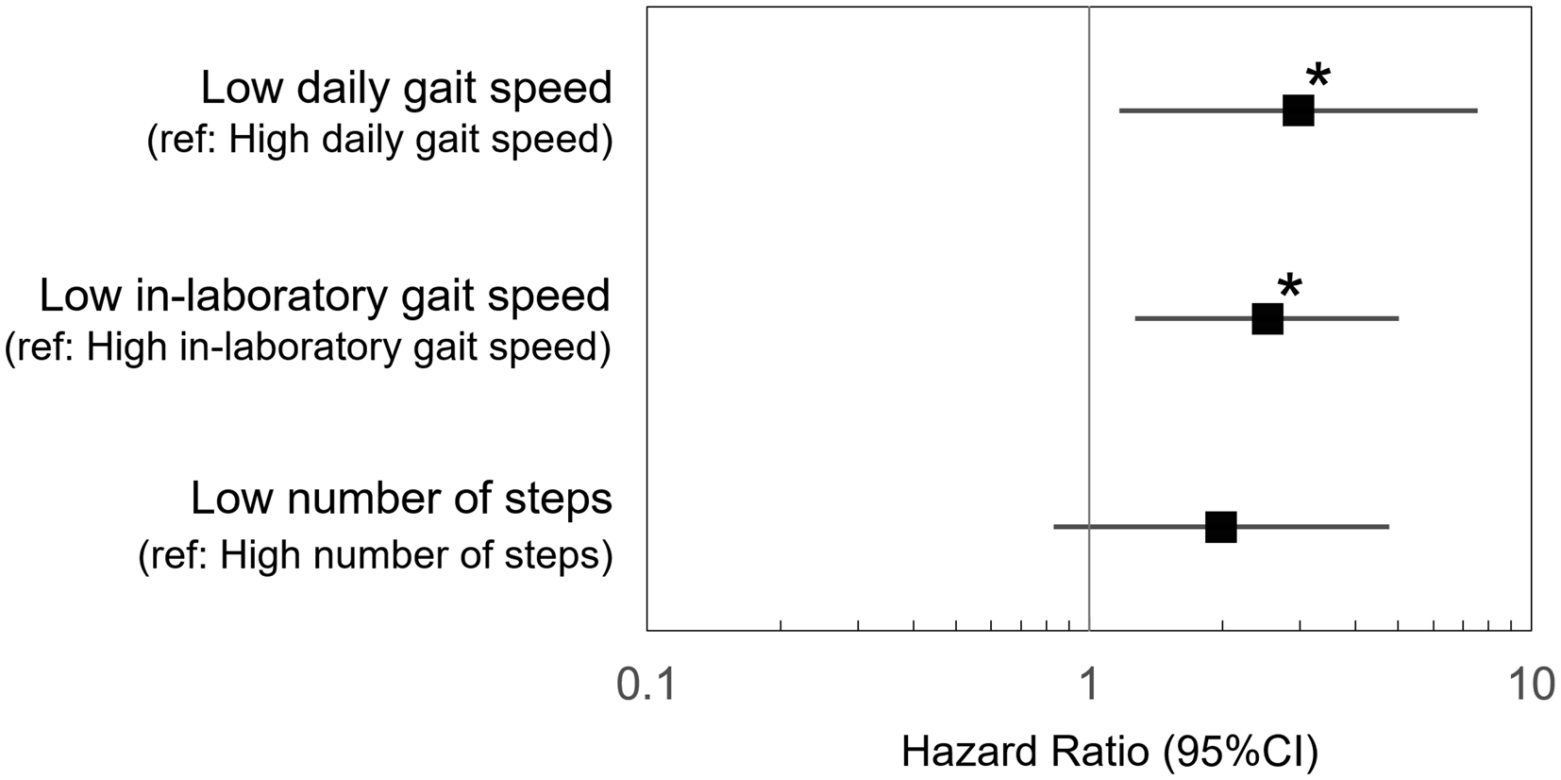
Hazard ratios (HRs) and 95% CIs for the three gait parameters (daily gait speed, in-laboratory gait speed, and number of steps) in the adjusted model. *p < 0.05.

In the adjusted model, the low daily gait speed group showed a significantly higher risk of incident disability than the high daily gait speed group, even after adjusting for covariates (HR, 2.97; 95% CI = 1.17–7.56; *p* = 0.02). Similarly, the low in-laboratory gait speed group showed a significantly higher risk of incident disability than the high in-laboratory gait speed group (HR: 2.53; 95% CI = 1.27–5.01; *p* = 0.01). Conversely, the low number of steps group showed no significant association with incident disability after adjustment for covariates (HR: 1.99; 95% CI = 0.83–4.78; *p* = 0.12). Figure 2 shows the HRs and 95% CIs for the three gait parameters and covariates in the adjusted model.

## Discussion

The objective of this study is to investigate whether daily gait speed can be a predictor of decline in physical function in older adults as well as in-laboratory gait speed. The association between daily gait speed with waist-worn accelerometers and incident disability during the two-year period were analyzed. In addition to daily gait speed, the number of steps with waist-worn accelerometers and in-laboratory gait speed were measured, and the association between these gait parameters and incident disability was also compared.

Cox proportional hazards analysis showed a significant hazard ratio of low daily gait speed (HR, 2.97; *p* = 0.02) comparable to that of low in-laboratory gait speed (HR: 2.53; *p* = 0.01). Conversely, for the number of steps, there was no significant association with incident disability (HR: 1.99; *p* = 0.12). A recent large cohort study revealed that in-laboratory gait speed is associated with incident disability risk^10^. Recently, measuring daily gait speed by using wearable sensors has been proposed^12–14^. However, there is only a weak correlation between daily gait speed and in-laboratory gait speed because of the difference in measurement conditions^17,21^. Therefore, in previous studies, it was not clear whether daily gait speed would be a useful predictor of the decline of physical function in older adults, such as in-laboratory gait speed. The present results suggest that daily gait speed can be a useful predictor of physical function decline in older adults. To the best of our knowledge, this is the first study to examine the relationship between daily gait speed and incident disability on large cohort data on community-dwelling older adults. The measurement of daily gait speed using an accelerometer is not restricted by place and can be done unconsciously, compared to in-laboratory gait speed measures. The present results suggest that by simply wearing accelerometers and allowing participants a free lifestyle, future functional declines in older adults can be predicted continuously and the need to maintain and improve physical function can be indicated beforehand.

Although the present study measured daily gait speed for older adults by using a waist-worn accelerometer, various conditions—such as device type, speed range, and measurement location on the body—are believed to affect the estimation accuracy of this parameter. For speed range, Soltani et al.^14^ compared the estimation algorithms for slow, normal, and fast walking, and operating walking aids using a lower-back-worn sensor and reported that different root mean square errors were obtained under these multiple conditions. Regarding location on the body, Zihajehzadeh et al.^22^ reported gait speed estimation using a wrist-worn sensor. Therefore, future studies are needed to verify whether the present findings can be adapted to other conditions such as device type, speed range, and measurement location on the body.

A previous study has shown that even physical activity or exercise behavior interventions alone have positive results in increasing physical activity among community-dwelling older adults^23^. Daily gait speed can be easily and continuously measured using an accelerometer. Therefore, it does not only detect the individual’s risk of incident disability at an early stage but also helps maintain motivation by allowing the older individuals and supporters to reflect on their results regularly. There is growing evidence that interventions that involve multiple behaviors may have a greater impact than those that involve a single behavior^24^. Therefore, daily gait speed can be measured and fed back, and, in addition, a further intervention effect can be expected by combining multiple actions, such as regular exercise, to increase physical activity.

To investigate the association between daily gait parameters (i.e., daily gait speed and number of steps) and incident disability at the two-year follow-up, we employed the cut-off values used in a previous study^18^ for screening older adults with prefrailty statuses. As prefrailty is reportedly a reversible state^25,26^, proactive interventions can reduce the progression to frailty. In the present study, the low daily gait speed group showed a significantly higher risk of incident disability than the high daily gait speed group. It is considered that the daily gait speed could predict incident disability during a two-year period, as a result of screening older adults with prefrailty status, who were at high risk of incident disability. Conversely, with respect to the number of steps, although the incidence of disability was significantly higher in the low number of steps group than in the high number of steps group, there was no significant association with incident disability after adjusting for covariates in the Cox proportional hazards analysis. Although a previous study has shown that the number of steps is significantly correlated with the progression of frailty^27^, this parameter was not suitable as a predictor from the viewpoint of predicting incident disability during the two-year period in the present analysis.

Furthermore, there were more low-speed groups of daily gait speed (n = 930) and low step group of the number of steps (n = 985) compared with the low-speed group of in-laboratory gait speed (n = 344) in the present study. The previous study has revealed that the daily gait speed was slower than the in-laboratory gait speed^17^. In the present study, using the cut-off values reported in previous studies^10,18^, the value of the daily gait speed (106.3 cm/s) was higher than that of the in-laboratory gait speed (100 cm/s). As this study was the first to investigate the relationship between daily gait speed and incident disability and the cut-off values from a previous study^18^ used, it is necessary to further examine the cut-off value in various regions and participants for daily gait speed to improve the risk prediction of incident disability.

The present study classified each of the three gait parameters (daily gait speed, in-laboratory gait speed, and the number of steps) into two groups based on the cut-off values, and compared them with the Kaplan–Meier curves. The incidence of disability during the two-year period was significantly higher in the groups with lower values compared with those with higher values in all three gait parameters. A previous study has reported that the model combining the daily gait speed and the number of steps had higher screening accuracy for older adults with prefrailty status than the model using a single parameter for the number of steps or the daily gait speed^18^. Furthermore, Bortone et al.^28^ performed a systematic review of the relationship between gait parameters and frailty, and stated that the combination of various gait parameters may enhance the prediction of frailty status and the classification of different frailty phenotypes. As mentioned above, although both daily gait speed and in-laboratory gait speed decreased with age^13,19^, there is only a weak correlation between the two gait speed parameters^17,21^. These insights indicate that these two gait speed parameters represent a physical decline from different perspectives. As in-laboratory gait speed is often measured over a short distance, it strongly shows aspects of individuals’ physical strength and physical function. Conversely, as daily gait speed is measured using an accelerometer in daily life, it indicates aspects of individuals' physical function as well as of their lifestyles. Therefore, by combining these parameters, it is possible to improve the prediction accuracy of future incident disability. Future studies should analyze these viewpoints to make predictions regarding older adults whose functional abilities have decreased with higher accuracy.

In the present study, 2.3% of all participants (age: 70.1 ± 6.2; n = 1860) experienced incident disability during the two-year period. In a previous study examining incident disability during the same period in Japanese older adults (age: 71.8 ± 5.4; n = 4341), 3.9% of participants experienced an incident disability^29^, where the incident disability was higher than in the present study. One of the factors is that participants included in the present analysis consisted only of those meeting the criteria to wear the accelerometers (i.e., they were wearing the accelerometer on their waist for a total duration of ≥ 7 days, for ≥ 10 h/day, during the first 14 days after the day they began wearing the accelerometer). Although many participants agreed to wear the accelerometers, they were unable to meet the criteria for obtaining accelerometer data in this study (n = 1941). The incident disability rate of these participants during the two-year period was 5.6% (n = 109), which resulted in a low incident disability rate in the present analysis (2.3%; n = 42). Therefore, the participants who did not meet the criteria for wearing accelerometers included older adults with physical decline, which might have been difficult for them to wear accelerometers. However, even with the exception of these participants, the Cox hazard analysis showed high HRs with daily gait speed and in-laboratory gait speed. These results suggest that even in the low-risk group, which does not include high-risk older adults whose physical dysfunction has become apparent, the daily gait speed and the in-laboratory gait speed can predict future incident disability risk.

This study has several limitations. First, this study was an analysis of a two-year follow-up where most of the participants were able to maintain their health functions, indicating a possible ceiling effect. A long-term follow-up study could reveal the long-term effects of daily gait speed and other gait parameters on the incidence of disability. Second, this study focused on older adults living in Takahama City, Aichi, Japan. As the amount of physical activity varies by country or region^30^, the cut-off values for the number of steps and daily gait speed are likely to change across populations. Therefore, to increase generalizability, future studies should compare national, regional, and/or cultural differences based on similar measurements. Third, the participants may have been motivated by wearing the accelerometer, thereby increasing their daily gait speed, which may have yielded inflated scores. Fourth, the number of prescribed medications and medical histories (of Parkinson’s disease, dementia, stroke, cancer, spine disease, fractures after age 60, osteoarthritis, heart disease, hypertension, diabetes, hyperlipidemia, osteoporosis, and respiratory disease) were collected through face-to face interviews with the participants in this study, which might have led to recall bias.

The present study analyzed the association between daily gait speed and incident disability during a two-year period. In addition, in-laboratory gait speed and number of steps were measured, and the screening accuracy was compared with the daily gait speed. In all three gait parameters, groups below the cut-off values resulted in significantly higher incident disability rates. Furthermore, the low daily gait speed and in-laboratory gait speed group showed high HRs. These results suggest that by simply wearing accelerometers and allowing free lifestyles, future functional decline in older adults can be predicted, and appropriate action can be taken to maintain and improve physical function before onset.

## Methods

### Participants

The present study is part of the National Center for Geriatrics and Gerontology Study of Geriatric Syndromes (NCGG-SGS), a cohort study aimed at establishing a screening system for geriatric syndromes^17,18^. A total of 4072 community-dwelling older adults (age ≥ 60 years) residing in Takahama City, Aichi, Japan, participated in the study. As in the previous studies^17,18^, all participants provided written informed consent by reading and signing a consent form approved by the institutional review board and agreed to wear accelerometers. We followed an opt-out approach, whereby we presented participants with the option to decline participation after providing them with information on the study. They could opt out directly or by proxy. This study was conducted in accordance with the guidelines of the Declaration of Helsinki. The study protocol was approved by the Research Ethics Committee of the National Center for Geriatrics and Gerontology (Approval Number 1440–2).

### Baseline assessments

Age, sex, self-reported educational history, the 15-item Geriatric Depression Scale (GDS) scores, and working, smoking, and alcohol drinking status were collected. GDS has been validated as a screening tool for depressive symptoms in older adults^31^. Grip strength was measured using a Smedley-type handheld dynamometer (Grip-D, Takei Ltd., Niigata). We measured the participants’ height and weight and calculated their Body Mass Index (BMI). Furthermore, licensed nurses recorded the number of prescribed medications and medical history regarding the following diagnoses in face-to-face interviews: Parkinson’s disease, dementia, stroke, cancer, spine disease, fractures after age 60, osteoarthritis, heart disease, hypertension, diabetes, hyperlipidemia, osteoporosis, and respiratory disease. A previous study has reported that the incidence of fractures is associated with disability, morbidity, and hospitalization, particularly in older adults^32^. Since the participants in this study were 60 years or older, the experience of fractures after age 60 was included at baseline assessments.

### In-laboratory gait speed measurement

In addition to the baseline assessment, a 6.4 m gait speed was defined as the in-laboratory gait speed as based on a previous study^17^. This parameter was measured using a sheet-type pressure sensor (2.4 m long; Walk Way, Anima Corporation, Tokyo, Japan) placed in the middle of a 6.4 m walkway, operating at a sampling frequency of 100 Hz. The participants were instructed to walk along the walkway at a comfortable pace. They repeated the 6.4 m walk five times, and the average in-laboratory gait speed was calculated.

### Daily data collection

As in the previous studies^17,18^, on the day of the baseline assessment, participants were instructed to wear one tri-axial accelerometer (HW-100, Kao Corporation, Tokyo, Japan) on their waists by inserting it into their pants’ pockets or their belts with a clip—at all times while awake, except when swimming or bathing—and to maintain their usual activities. Participants were instructed to wear this device for at least 14 days starting the day after the baseline assessments, and to visit one of 75 designated locations in Takahama City, where the accelerometer data were downloaded to a tablet PC via a Near Field Communication (NFC) system (Sony Corporation, Tokyo, RC-380). Designated locations were chosen for their ease of access, and included government-run facilities (e.g., community centers), gyms, drug stores, cafeterias, and beauty salons^17,18^.

### Daily gait speed and number of steps

The daily gait speed and number of steps were measured using an accelerometer (HW-100) to ensure continuous monitoring during daily living^17^. HW-100 is a tri-axial accelerometer providing 40 days of continuous recording at a sampling frequency of 64 Hz, with ± 2 g range. The number of steps is calculated based on the composite acceleration from tri-axial acceleration. A low-pass filter is applied to the composite acceleration, and the differential value of this acceleration that occurs during walking is monitored. The device records consecutive steps of seven or more from the start of walking as number of steps. Data for 14 days from the day after the participants wore the accelerometer were included in the analysis. In addition, the device also monitors acceleration in the direction of gravity every four seconds based on tri-axis acceleration and defines the wearing time as the time excluding when this acceleration was not continuously recorded for more than 35 min^33^. A valid day was defined as any day on which the accelerometer was worn for ≥ 10 h, and participants with fewer than seven valid days were excluded from the analysis, according to a systematic review^34^.

### Algorithm for daily gait speed estimation

This device (HW-100) detects gait cycle during gait ranging from 70 to 160 steps/min by medio-lateral and vertical acceleration. The recording of tri-axial acceleration in a gait cycle is initiated if the current cycle values and the two preceding cycle values are within 10% of each other. In other words, since the device starts recording acceleration from the third gait cycle at the earliest, acceleration during only steady-state periods of gait can be detected. This recording continues until the current gait cycle value deviates from this range. As a result, the average acceleration on the three-axes during one gait cycle per day is calculated. Daily gait speed was calculated with a model that used composite acceleration from the average tri-axial acceleration during one gait cycle.

A previous study reported on the accuracy of gait speed measured with this device, which was attached to individuals’ waists^17^. Forty-six participants (40.8 ± 10.9 years old) were instructed to walk along the walkway twice—at three kinds of pace each; (1) usual pace, (2) faster than usual pace, and (3) slower than usual pace; the gait speed was measured at 20 m during steady-state periods of gait with a stopwatch as the gold standard. This study reported a strong correlation with gait speed measured by this device (*r* = 0.848, *p* < 0.001). However, this study also reports that the device had a systematic error of 14.3 cm/s compared to the gold standard, which suggests that it may underestimate the daily gait speed. Therefore, daily gait speed was adjusted by adding 14.3 cm/s to the observed value in the present study.

### Incident disability

Participants were followed-up for incident certification for the need of care according to the Japanese LTCI system^6^ during the two years after the baseline assessment. This system uses a statistical model developed in a large time study to estimate the level of care needed regarding nursing care minutes based on an assessment of physical and mental status^6^. First, a local government official conducts an interview regarding their current physical and mental condition (including physical function, activities of daily living (ADL), cognitive function, behavioral and psychological symptoms, and social adaptation ability) and their history of medical procedures. From the results of this questionnaire, the time required for nursing care is predicted to determine the necessity of temporary nursing care. Subsequently, the Nursing Care Needs Certification Board reviews and confirms the care need level: “Support Level 1 or 2” to indicate a need for assistance to support ADL or “Care Level 1 through 5” to indicate a need for continuous care. In the present study, incident disability was defined as the point at which a participant was certified as needing care according to the LTCI classification, regardless of the care level.

### Data analysis

Participants were excluded from the statistical analysis if they (1) had a history of Parkinson’s disease (n = 18), (2) had dementia (n = 8), (3) had missing data for any of the baseline assessments (n = 164), or (4) were lost to follow-up during the two years after baseline assessment (n = 81). Furthermore, based on the previous studies^17,18^, we also excluded participants who were unable to meet the criteria for accelerometer data (n = 1941), which means that they (i) did not go to one of the designated locations within 60 days after they started wearing the accelerometer, or (ii) did not wear the accelerometer on their waist for a total of ≥ 7 days for ≥ 10 h/day, during the first 14 days after starting to wear the accelerometer. A total of 1860 participants (45.7%) were included in the final analysis. On average, participants wore the accelerometer for 11.8 ± 2.2 days, with an average wearing time of 14.2 ± 1.9 h/day.

Based on the cut-off values of previous studies^10,18^, each of the three gait parameters was classified into two groups. Regarding daily gait speed, participants in the low daily gait speed group had speed < 106.3 cm/s (n = 930), and the high daily gait speed group were participants with a speed ≥ 106.3 cm/s (n = 930). Regarding in-laboratory gait speed, the low in-laboratory gait speed group included participants with a speed < 100.0 cm/s (n = 344), and high in-laboratory speed group were participants with a speed ≥ 100.0 cm/s (n = 1516). Regarding number of steps, the low number of steps group included participants with steps < 6342.2 steps/day (n = 985) and the high number of steps group were participants with steps ≥ 6342.2 steps/day (n = 875).

### Statistics

Unpaired *t*-tests and chi-squared tests were conducted to test differences in baseline characteristics and three gait parameters (daily gait speed, in-laboratory gait speed, and number of steps) between participants with incident disability during the two years after baseline assessment and those without. Furthermore, the relationships between the three gait parameters were examined by calculating Pearson’s correlation coefficients (*r*). The cumulative incidence of disability rate according to the three gait parameters during the follow-up period were assessed using the Kaplan–Meier curve, and the differences were examined using the log-rank test. Cox proportional hazards regression models were used to analyze the associations between the three gait parameters and disability risk. The HRs and 95% CIs of disability were calculated in the crude and adjusted models (adjusted variables were age, sex, BMI, grip strength, education years, GDS score, number of prescribed medications, working, smoking, alcohol drinking, stroke, cancer, spine disease, fractures after age 60, osteoarthritis, heart disease, hypertension, diabetes, hyperlipidemia, osteoporosis, and respiratory disease). Cohen’s *d* and the phi coefficient (*φ*) are reported as effect sizes^35^. Differences in means were considered statistically significant when *p*, *d*, and *φ* values were less than 0.05, greater than 0.20, and greater than 0.10, respectively. All statistical analyses were conducted using Python version 3.6.13. Unpaired *t*-tests and chi-squared tests were conducted using the Python package SciPy (version 1.6.2)^36^. Kaplan–Meier analysis, log-rank test, and Cox proportional hazards regression analysis were conducted using the Python package lifelines (version 0.26.0)^37^.

## Data Availability

The datasets generated and/or analyzed during the current study are not publicly available because of intellectual property reasons, but are available on reasonable request.
